# Pharmaceutical Agents as Potential Drivers in the Development of Early-Onset Colorectal Cancer: Case-Control Study

**DOI:** 10.2196/50110

**Published:** 2023-12-13

**Authors:** Irit Ben-Aharon, Ran Rotem, Cheli Melzer-Cohen, Gilad Twig, Andrea Cercek, Elizabeth Half, Tal Goshen-Lago, Gabriel Chodik, David Kelsen

**Affiliations:** 1 Department of Gastroenterology Rambam Healthcare Campus Haifa Israel; 2 Harvard T Chan School of Public Health Boston, MA United States; 3 KSM Research and Innovation Center Maccabi Healthcare Services Tel-Aviv Israel; 4 The Institute of Endocrinology Diabetes and Metabolism Sheba Medical Center Ramat Gan Israel; 5 Department of Preventive Medicine and Epidemiology, School of Public Health Sackler Faculty of Medicine Tel Aviv University Tel Aviv Israel; 6 Gertner Institute for Epidemiology & Health Policy Research Sheba Medical Center Ramat Gan Israel; 7 Gastrointestinal Oncology Service Department of Medicine Memorial Sloan Kettering Cancer Center New York, NY United States; 8 Weill Cornell Medical College New York, NY United States

**Keywords:** early onset colorectal cancer, pharmaceutical agents, increased risk, colorectal cancer, health provider, Israel, machine learning, decision tree, gradient boosting, risk factors, decision support, risk, risks, colorectal, cancer, oncology, internal medicine, gastroenterology, gastrointestinal, pharmaceutical, pharmaceuticals, drug, drugs

## Abstract

**Background:**

The incidence of early-onset colorectal cancer (EOCRC) rose abruptly in the mid 1990s, is continuing to increase, and has now been noted in many countries. By 2030, 25% of American patients diagnosed with rectal cancer will be 49 years or younger. The large majority of EOCRC cases are not found in patients with germline cancer susceptibility mutations (eg, Lynch syndrome) or inflammatory bowel disease. Thus, environmental or lifestyle factors are suspected drivers. Obesity, sedentary lifestyle, diabetes mellitus, smoking, alcohol, or antibiotics affecting the gut microbiome have been proposed. However, these factors, which have been present since the 1950s, have not yet been conclusively linked to the abrupt increase in EOCRC. The sharp increase suggests the introduction of a new risk factor for young people. We hypothesized that the driver may be an off-target effect of a pharmaceutical agent (ie, one requiring regulatory approval before its use in the general population or an off-label use of a previously approved agent) in a genetically susceptible subgroup of young adults. If a pharmaceutical agent is an EOCRC driving factor, regulatory risk mitigation strategies could be used.

**Objective:**

We aimed to evaluate the possibility that pharmaceutical agents serve as risk factors for EOCRC.

**Methods:**

We conducted a case-control study. Data including demographics, comorbidities, and complete medication dispensing history were obtained from the electronic medical records database of Maccabi Healthcare Services, a state-mandated health provider covering 26% of the Israeli population. The participants included 941 patients with EOCRC (≤50 years of age) diagnosed during 2001-2019 who were density matched at a ratio of 1:10 with 9410 control patients. Patients with inflammatory bowel disease and those with a known inherited cancer susceptibility syndrome were excluded. An advanced machine learning algorithm based on gradient boosted decision trees coupled with Bayesian model optimization and repeated data sampling was used to sort through the very high-dimensional drug dispensing data to identify specific medication groups that were consistently linked with EOCRC while allowing for synergistic or antagonistic interactions between medications. Odds ratios for the identified medication classes were obtained from a conditional logistic regression model.

**Results:**

Out of more than 800 medication classes, we identified several classes that were consistently associated with EOCRC risk across independently trained models. Interactions between medication groups did not seem to substantially affect the risk. In our analysis, drug groups that were consistently positively associated with EOCRC included beta blockers and valerian *(Valeriana officinalis*). Antibiotics were not consistently associated with EOCRC risk.

**Conclusions:**

Our analysis suggests that the development of EOCRC may be correlated with prior use of specific medications. Additional analyses should be used to validate the results. The mechanism of action inducing EOCRC by candidate pharmaceutical agents will then need to be determined.

## Introduction

An abrupt increase in the incidence of early-onset colorectal cancer (EOCRC), generally defined as the occurrence of colon or rectal cancer in people less than 50 years of age, began in the mid 1990s; this was first noted in the early 2000s [1-3]. The majority of EOCRC cases are not due to inherited cancer susceptibility genes or inflammatory bowel disease (IBD), which have long been known as risk factors for EOCRC [3-5]. This implies that the driving factors for the increase in EOCRC are environmental, although there is presumably an interplay between genes and the environment. The rise in the incidence of EOCRC has now been documented in many countries [3,6,7]. While an increasing prevalence of obesity, high processed foods diets, diabetes, smoking, alcohol consumption, and sedentary lifestyles have been implicated in EOCRC, these lifestyle and comorbidity factors have been present since the 1950s, and the marked increase in EOCRC was noted beginning in the 1990s [8,9]. Further, many patients with EOCRC are neither obese nor sedentary; data on obesity as an increased risk factor for EOCRC report both increased and decreased risk compared to controls [10-13]. The extensive use of antibiotics has also been suggested as a causative factor through the induction of changes to the spectrum of the gut microbiome, but data regarding the gut microbiome and metabolites in patients with EOCRC compared to unaffected patients are conflicting [14,15]*.*

The observation that the increase in EOCRC incidence was abrupt rather than occurring gradually over decades suggested to us that a factor leading to colonic neoplasia that was not previously widely used was made available to young people over a relatively short period of time. We thus hypothesized that a driver for EOCRC could be an off-target effect of a medication that acts directly or indirectly on the colonic mucosa to result in the activation of oncogenic pathways or silencing of protective pathways in individuals who otherwise have a benign genetic makeup. These medications were either not previously available or not previously widely used and were prescribed by physicians or obtained over-the-counter (OTC) by young people. These medications could be new drug approvals by regulatory agencies through new drug applications (NDAs) or supplementary NDAs or drugs that began to be used for uses other than the approved indication (ie, off-label use). The pharmaceutical agent could act either directly on the colonic mucosa or indirectly (eg, via the gut microbiome or its metabolites). Even though EOCRC is increasing in incidence, it still affects only a small fraction of all young people. Young patients developing EOCRC may have a genetic predisposition, such as a genetic polymorphism (possibly resulting in altered drug metabolism), which translates into an off target carcinogenic effect.

To test our hypothesis, we sought to identify pharmaceutical agents used more frequently in patients with EOCRC than in their peer controls and determine which may be risk factors for EOCRC by using a novel machine learning method to analyze a large Israeli electronic medical records (EMRs) database that includes digitized pharmacy records for patients with EOCRC and matched controls.

## Methods

### Study Population

To identify pharmaceutical agents related to EOCRC, a nested case-control study was performed using data from Maccabi Healthcare Services (MHS), an Israeli state-mandated health provider that serves 2.6 million members (26% of the Israeli population). EMRs have been used in MHS since the mid 1990s. Each patient’s EMR contains individual-level demographic and clinical information, including clinical diagnoses, hospitalizations, medical procedures, laboratory test results, and filled prescriptions.

### Case Ascertainment and Matching

MHS maintains a cancer registry though linkage with the Israel National Cancer Registry (INCR). The INCR was established in 1965 with the aim of continuously collecting data on newly diagnosed cancer cases from all medical institutions in Israel. Since 1981, all pathology results from diagnosed cancer cases must be submitted to the INCR. To further ensure complete case ascertainment, the MHS cancer registry supplements the national database with 2 additional sources of data, namely, histological findings from the MHS central laboratory and purchase authorization records for cancer-specific medications.

We used the MHS cancer registry to retrieve information on all patients with colorectal cases first diagnosed between January 1, 2001, to December 31, 2019, who received their first cancer diagnosis at age 50 years or younger (n=1461). To minimize misclassification of medication exposure, we restricted the analysis to patients who were continuously enrolled in MHS for at least 3 years prior to the index date (n=1174). We also excluded 237 cases who had EMR indications of IBD (Crohn disease or ulcerative colitis) or a personal or family history of suspicion for an inherited cancer susceptibility gene. Thus, the final case population included 941 patients with colorectal cancer.

For each case we matched 10 controls through density (risk-set) matching. Controls had to be continuously enrolled in MHS for at least 3 years before the index date, have no personal history of colorectal cancer, and have no evidence of colorectal cancer at the index date. Matching was done using sex, residential district, sociodemographic status (based on enumeration area, which is the smallest geostatistical unit of the Israeli central bureau of statistics), minority subpopulation at the residential area (Israeli Arab or Jewish Orthodox and Ultra Orthodox or Jewish secular), birth year (±2 years), and periphery index (proximity to a large urban center).

### Exposure Assessment

MHS members can fill prescriptions at any of more than 700 affiliated pharmacies across the country with a minimum copayment. OTC medications can also be purchased through these pharmacies at a discount. For both cases and controls, we obtained information on all dispensing of medications (OTC and prescription) prior to the index date, implementing a 2-year lag prior to the index date to reduce the likelihood of reverse-causation. To reduce the dimensionality of the data and ease interpretation, individual medications were grouped according to the fifth level (chemical substance) of the Anatomical Therapeutic Chemical classification system. Drugs that were used by less than 0.1% (103/10,351) of the cohort were excluded.

### Comorbidity Information

Our goal in this analysis was to identify medication classes that were linked with EOCRC to inform future studies. Thus, we did not aim to make casual interpretations. We collected information on several comorbidities recorded before the index state that have been linked with EOCRC [16-18]. These included diabetes (International Classification of Diseases, Ninth Revision [ICD-9] code 250.x); hypertension (ICD-9 code 401.x); cardiovascular conditions including ischemic heart disease (ICD-9 codes 410.x, 412, 429.7, and 429.79), non–myocardial infarction (ICD-9 codes 36.x, 411.x, 414.x, and 429.2), congestive heart failure (ICD-9 codes 404.x and 428.x), peripheral vascular disease (ICD-9 codes 440.x, 441.x, 442.x, and 443.x), stroke (ICD-9 codes 433.x1 and 438.x), transient ischemic attack (ICD-9 code 435.x), cerebrovascular atherosclerosis (ICD-9 code 434.x0), and atrial fibrillation (ICD-9 code 427.3x); and obesity (ICD-9 code 278.02). Data on comorbidities were obtained from the MHS automated patient registries, which apply case ascertainment algorithms to define patients with chronic diseases according to multiple data sources and disease-specific international guidelines. These include separate registries for cardiovascular disease, diabetes, and hypertension. MHS physicians are required to measure patients’ body weight annually and document this in the EHR [17-20]. To assess potential surveillance bias, we also calculated the average frequency of monthly physician visits for each patient by summing all physician visits recorded for a given patient by each patient’s follow-up time in the system up to 2 years before the index date, as this can affect the likelihood and timing of receiving an EOCRC diagnosis as well as the nature of medication prescription patterns.

### Statistical Analysis

The very high dimensionality of the medication dispensing data limits the use of conventional statistical methods for the analysis since such methods cannot account for the frequent coexposure to many different medication classes. Thus, to conduct a high-throughput screening of medications and identify specific medication groups, individual or in combination, that were consistently linked with EOCRC, we used eXtreme Gradient Boosting (XGBoost), a highly efficient implementation of gradient tree boosting [21]. Briefly, gradient boosting is an ensemble technique that combines the outputs of many individual decision trees to capture complex associations with an outcome of interest. The combining of trees is done iteratively so that each new tree is fit on the residuals of the previous one. XGBoost uses parallel processing to reduce computing time and advanced regularization to enhance the stability of the predictions. The model requires defining several data-specific regularization parameters to optimize performance, which were selected though a 5-fold cross-validation coupled with a Bayesian model optimization [22].

To enhance the reproducibility of our findings, we trained 50 independent XGBoost models, each time setting aside a randomly selected 20% of the data. A list of medication groups associated with EOCRC was derived through global Shapley additive explanations (SHAP) values [23], focusing on medication groups that were associated with the outcome in at least 50% of the independent runs or those with global SHA*P* value in the top 2.5% of the distribution in at least 1 of the runs. A final XGBoost model was then trained with these selected medication groups using the full data to evaluate the presence of informative interactions, assessed through the tree depth hyperparameter following final model optimization. Since machine learning models, including XGBoost, do not provide specific effect estimates, the final list of medications was then included in a conditional logistic regression model to obtain odds ratios (ORs) with 95% CIs for each medication category while accounting for the matched risk sets and adjusting for the frequency of physician visits as well as the patients’ history of diabetes, cardiovascular disease, and excess body weight at baseline that were found to be significant in the multivariable model. All statistical analyses were performed using R (version 4.0.2, R Foundation for Statistical Computing).

### Ethical Considerations

The study protocol was approved by the MHS institutional review board (MHS IRB 0034-24). Informed consent was waived by the institutional review board because patients’ identifying details were removed. There was no compensation provided to individual patients for participation in the study.

## Results

Population characteristics are shown in Table 1. The average age at the time of EOCRC diagnosis was 43.6 (SD 6.7) years. The average number of follow-up months and the frequency of physician visits was similar for cases and controls. Cases and controls were well-matched.

Of the more than 800 medication classes used by the study participants, we identified 5 classes that were consistently associated (>50%) with EOCRC risk across the independently trained models. An additional 10 medication groups not consistently associated with EOCRC also had SHA*P* values in the top 2.5% in at least 1 run (Table 2). Interactions between medication groups did not seem to be informative for risk prediction.

An examination of the effect estimates for the identified medication groups from the conditional regression model adjusted for the aforementioned comorbidities suggested several medication classes whose use was associated with increased odds of EOCRC. These included a beta-blocker, an angiotensin converting enzyme (ACE) inhibitor, and valerian, an herbal medication. Some protective associations were also observed, including for paroxetine, a selective serotonin uptake inhibitor. Antibiotics were not consistently associated with increased risks (Table 3).

**Table 1:** Demographics of patients with colorectal cancer and controls from data covering the period 2001-2019.

Demographics		Colorectal cases (n=941)	Controls (n=9410)
**Sex, n (%)**			
	Male	459 (48.8)	4590 (48.8)
	Female	482 (51.2)	4820 (51.2)
**Population subgroup, n (%)**			
	Secular Jewish	829 (88.1)	8290 (88.1)
	Jewish Ultra Orthodox	64 (6.8)	640 (6.8)
	Israeli Arab	48 (5.1)	480 (5.1)
**Residential area, n (%)**			
	North	176 (18.7)	1760 (18.7)
	Center	183 (19.4)	1830 (19.4)
	Jerusalem	211 (22.4)	2110 (22.4)
	Sharon	212 (22.5)	2120 (22.5)
	South	159 (16.9)	1590 (16.9)
Sociodemographic status^a^, mean (SD)		6.5 (1.9)	6.5 (1.9)
Weeks of follow-up before index date, mean (SD)^a^		641 (273)	646 (278)
Age at index date^b^ (years), mean (SD)		43.6 (6.7)	43.6 (6.6)
Physician visits over follow-up period^c^, yearly mean (SD)		7.2 (6.0)	7.2 (6.0)

^a^Sociodemographic status was measured on a 10-point scale (1: lowest; 10: highest).

^b^The index date was defined as the first date of colorectal cancer diagnosis.

^c^Used as a proxy measure of the frequency of contact with the medical system, which could affect the likelihood and timing of receiving a colorectal cancer diagnosis and medication prescription patterns.

**Table 2:** Prevalence of the use of pharmaceutical agents in patients with EOCRC and controls using eXtreme Gradient Boosting from data covering the period 2001-2019.

ATC^a^ class	ATC description	Prevalence of use in cases, n (%)	Prevalence of use in controls, n (%)	Rate of runs in which an association with CRC^b^ was observed^c^ (%)	Rate of runs with a global SHAP^d^ value ≥97.5% of the distribution^e^ (%)
c07aa05	Propranolol	42 (4.5)	228 (2.4)	70	5
c09aa02	Enalapril	44 (4.7)	161 (2.8)	60	3
n05cm09	Valerian	47 (5)	291 (3.1)	58	2
n06ab05	Paroxetine	15 (1.6)	376 (4)	54	10
c10aa01	Simvastatin	99 (10.5)	724 (7.7)	50	4
a10ba02	Metformin	40 (4.3)	234 (2.5)	46	2
d04aa13	Dimetindene	67 (7.1)	906 (9.6)	40	1
s01ca01	Dexamethansone	175 (18.6)	1986 (21.1)	36	3
d07ac13	Mometasone	103 (10.9)	1249 (13.3)	32	1
d11ax18	Diclofenac	86 (9.1)	672 (7.1)	30	1
a02ba03	Famotidine	161 (17.1)	1381 (14.7)	28	1
s01aa01	Chloramphenicol	301 (32)	2782 (29.6)	24	2
j01ce02	Phenoxymethylpenicillin	335 (35.6)	3443 (36.6)	20	1
r01ba52	Pseuroephedrine, combination	243 (25.8)	2631 (28)	20	1
b03ad01	Ferrous amino acid complex	174 (18.5)	1590 (16.9)	16	1
g01aa10	Clindamycin	79 (8.4)	685 (7.3)	10	1
r03ac03	Terbutaline	51 (5.4)	451 (4.8)	2	1

^a^ATC: Anatomical Therapeutic Chemical classification system.

^b^CRC: colorectal cancer.

^c^Based on global SHA*P* values calculated for each of 50 runs. A global SHA*P* value >0 for a given feature was considered evidence for an association with the outcome.

^d^SHAP: Shapley additive explanation.

^e^Considering each of the 50 different global SHA*P* values distributions individually.

**Table 3:** Odds ratios considering each of the 50 different global Shapley additive explanation value distributions from data covering the period 2001-2019.

ATC^a^ class	ATC description	Odds ratio^b^ (95% CI)	*P* value
c07aa05	Propranolol	1.94 (1.37-2.77)	<.001
n05cm09	Valerian	1.61 (1.15-2.25)	.01
c09aa02	Enalapril	1.34 (0.9-1.98)	.15
d11ax18	Diclofenac	1.33 (1.04-1.71)	.03
c10aa01	Simvastatin	1.3 (1-1.69)	.05
g01aa10	Clindamycin	1.22 (0.94-1.59)	.14
s01aa01	Chloramphenicol	1.19 (1.02-1.4)	.03
a02ba03	Famotidine	1.17 (0.97-1.42)	.10
r03ac03	Terbutaline	1.17 (0.86-1.59)	.32
b03ad01	Ferrous amino acid complex	1.15 (0.95-1.4)	.16
a10ba02	Metformin	1.14 (0.66-1.96)	.65
j01ce02	Phenoxymethylpenicillin	0.96 (0.82-1.11)	.58
r01ba52	Pseuroephedrine, combination	0.86 (0.73-1.02)	.07
s01ca01	Dexamethansone	0.8 (0.67-0.97)	.02
d07ac13	Mometasone	0.79 (0.63-0.98)	.04
d04aa13	Dimetindene	0.71 (0.54-0.92)	.01
n06ab05	Paroxetine	0.33 (0.2-0.57)	.001

^a^ATC: Anatomical Therapeutic Chemical classification system.

^b^Effect estimates from conditional logistic regression accounting for the matched risk sets, with additional adjustment for the average frequency of physician visits and any history of diabetes, cardiovascular disease, or excess weight prior to the index date.

## Discussion

### Principal Results

Our analysis of the use of pharmaceutical agents based on data from a quarter of the Israeli population during the period from 2001 to 2019, shortly after the marked increase in EOCRC incidence began, identified several medications that were used significantly more frequently by patients affected by early-onset colorectal cancer than their age-matched controls. Antibiotics, proposed in other studies as the responsible agent for the increase in EOCRC cases by changing the gut microbiome, were not used more frequently in patients with EOCRC than in controls. Agents used more frequently in patients with EOCRC included beta-blockers, ACE inhibitors, and an herbal supplement, valerian.

### Comparison With Other EOCRC Research Studies

The incidence of EOCRC has steadily increased since the mid 1990s; it is estimated that of the approximately 153,000 new cases of colorectal cancer diagnosed in 2023 in the United States, 19,550 will be in people younger than 50 years of age [24,25]. By 2030, it is estimated that 1 in 10 new cases of colon cancer and 1 in 4 cases of rectal cancer will occur in young people. EOCRC is on the rise in many countries [7,26].

The abrupt increase in the incidence of EOCRC suggests that the causative agent is an environmental factor, as neither IBD colitis-associated cancers nor inherited cancer susceptibility genes have increased in incidence, and neither are found in the large majority of patients with EOCRC [3,4,27,28]. The continued increasing incidence of EOCRC further suggests that the environmental factor is still present; while the absolute number of cases is small, age cohort analyses suggest the increasing incidence is higher in younger age cohorts (eg, 20-29 years of age) and that succeeding age cohorts have a higher incidence of EOCRC [29-31]. The large majority of EOCRC cases are located on the left side of the colon, especially in the rectum [32,33]. While some studies have shown little difference in the spectrum of tumor genomic alterations between EOCRC and sporadic cancer in older patients, others have not [4,34]. A recent analysis noted differences based on gender and ethnicity in nonhypermutated tumors in patients with EOCRC [4,34,35]. However, these somatic tumor genomic analyses did not focus on the initiating driving factors in “normal” colonic mucosae.

The suspicion that an environmental factor is responsible for the increasing incidence of EOCRC has led to analyses of comorbidities including obesity, diabetes mellitus, and lifestyle factors, such as a sedentary rather than active lifestyle, smoking and alcohol use, and the widespread use of antibiotics leading to a change in the gut microbiome [8,13,15]. For alcohol use, a recent population-based study from South Korea found an association between moderate and heavy alcohol use and an increased incidence of left-sided colon and rectal cancers, but not proximal cancers [36]. The effect was greater in men than in women. However, alcohol use was self-reported and assessed at a single point, not over time, and comorbidities including IBD and a family history of colorectal cancer were not excluded. For other environmental factors, such as obesity, some studies have found an increased EOCRC risk, but others have not [10-12]. Factors such as obesity, diabetes mellitus, more sedentary lifestyles, smoking, and alcohol use have increased gradually over decades and do not address the abrupt increase in the incidence of EOCRC.

The sharp increase in incidence suggests that a new inciting factor was introduced to a target population. This led us to hypothesize that a medication not previously widely used in young people had been introduced into general use and either directly or indirectly initiates neoplasia in colonic mucosa. A medication may have been introduced either through regulatory approval (NDAs), approval for an additional indication for an already approved drug (supplementary NDAs), a change in practice in which an already approved agent is more widely used by physicians to treat a condition other than the approved indication, or a new use of an OTC agent. While the incidence of EOCRC is increasing, it still affects only a small percentage of people under the age of 50 years. We therefore further hypothesize that in younger people who develop EOCRC, an otherwise benign genetic variant (not resulting in colonic neoplasia in the absence of exposure to the suspected medication) is potentiated by exposure to the medication to initiate or propagate neoplasia.

In order to obtain data for an initial evaluation of our hypothesis, it is necessary to have access to both detailed demographic and clinical information linked to detailed medication use data. In Israel, all citizens have access to health care through 4 health maintenance organizations. Detailed and extensive demographic, clinical, and pharmaceutical data are available to allow an analysis of pharmaceutical agents and clinical outcomes. The databases of Israeli health maintenance organizations have enabled the assessment of the risk of vaccine toxicities during the COVID-19 pandemic, evaluating, for example, the incidence and patients affected by myocarditis [37]. For our study, we used digitized records including pharmaceutical use both of prescribed and OTC agents from a large segment of the Israeli population as the source for our data. Our analysis indicated that, as we hypothesized, the use of several pharmaceutical agents was more common in young people diagnosed with EOCRC when compared to their control peers.

In regard to the agents that we have identified in our initial analysis, for propranolol, an agent one would not ordinarily expect to be prescribed to young people, we suspect its use was to treat anxiety or for side effects of antianxiety agents. Antihypertensives, such as ACE inhibitors, may be prescribed more commonly in young people as both stress and other conditions, such as obesity, increase the risk for hypertension. Both propranolol and valerian have been reported to have suppressive effects on established cancer cells; to our knowledge, there are no data as to their effects in inducing dysplasia and cancer in normal colon mucosae cells [38-40]. Data concerning ACE inhibitors and the risk for cancer are conflicting, with a recent report indicating null associations between use of ACE inhibitors and colorectal cancer risk [41]. Previous studies have suggested an association between pharmaceutical agents and cancer, including insulin use and breast cancer, antihypertensives and skin cancer, and sitagliptin and pancreas cancer, but these associations have not yet been confirmed. Yang et al [42] recently reported a web-based algorithm to assess cancer risk for 6 classes of pharmaceuticals (including antihypertensives, antihyperuricemics, antihyperlipidemics, nonsteroidal anti-inflammatory drugs, and antianxiety agents) in Taiwanese patients. However, the study only included agents used within 2 years of cancer diagnosis, which is a period during which the cancer may have already developed. For EOCRC, only 4 patients were between ages of 20-39 years, and the number of patients younger than 50 years was not provided.

The agents we have identified in our initial analysis do not include antibiotics. While it has been suggested that the widespread use of antibiotics during childhood and adolescence may underlie a carcinogenic effect on the gut microbiome, which may be translated into an increased incidence of EOCRC, the data are conflicting. In a recent British study, antibiotic consumption was associated with colorectal cancer in both older and EOCRC cohorts, while a population-based case-control study failed to find conclusive evidence that antibiotics were associated with EOCRC risk [43,44]. In a population-based analysis of the effect of cesarian section (removing exposure to the maternal vaginal flora), while a trend for a higher rate of EOCRC in women born by vaginal delivery was found, the overall analysis did not show a difference in the incidence of EOCRC in individuals born by cesarian section versus those undergoing vaginal delivery [45].

If our hypothesis is correct and validation studies confirm that a pharmaceutical agent or class of agents is a driver in the development of EOCRC, this would lead to regulatory bodies requiring strategies to mitigate risk [46]. Guidance to industry from the US Food and Drug Administration, for example, provides instructions for determining the level of risk, the patient populations at risk, and when available, biomarkers to determine if an individual patient is at risk. If a companion diagnostic test (eg, a genetic polymorphism found in patients with EOCRC but not in their unaffected peers) is developed, this would be included in the labeling indication. Examples of risk mitigation strategies for other illnesses include labeling indication instructions to identify patients with CYP2C19 alleles that poorly or almost totally do not metabolize clopidogel (Plavix) to minimize the risk of using an ineffective drug or drug dose [47]. For thalidomide, no safe dose has been identified for use of this agent in women who are pregnant, and the labeling indication includes a black box warning.

### Strengths and Limitations

Strengths of our study include the use of an EMR including digitized pharmaceutical data covering a large percentage of an entire country’s population, long-term follow-up, and the use of an advanced machine learning algorithm. We recognize that while we used extensive matching and adjustment for possible confounders, as well as repeated sampling of the data to enhance reproducibility, our results are still susceptible to confounding by indication. That is, rather than reflecting an association with the medications, the observed finding could reflect associations with the underlying conditions for which the medications were prescribed [48]. We also recognize the sample size limitations and have not drawn causality conclusions regarding the agents identified as being used more frequently in patients with EOCRC compared to controls. To assess the risk of publication bias, we systematically reviewed the literature with regard to our hypothesis that a pharmaceutical agent is a driving factor in the abrupt increase in the incidence of EOCRC and found no publications to date that have studied this hypothesis specifically in EOCRC. We regard our current data as supporting our hypothesis but note that validation studies from similar health care system databases are needed. Moreover, prospective studies to identify the mechanism of action of pharmaceutical agents used more frequently by patients with EOCRC than controls on normal colonic mucosae in initiating a neoplastic process in a susceptible population of younger adults are required. We have initiated such studies.

In conclusion, our analysis suggests that EOCRC may be associated with the prior use of specific medications. Additional studies of pharmaceutical agents as possible drivers of EOCRC and the potential mechanisms of action for this effect are underway.

## Abbreviations

ACE: angiotensin converting enzyme
EMR: electronic medical record
EOCRC: early-onset colorectal cancer
IBD: inflammatory bowel disease
ICD-9: International Classification of Diseases, Ninth Revision
INCR: Israel National Cancer Registry
MHS: Maccabi Healthcare Services
NDA: new drug application
OR: odds ratio
OTC: over-the-counter
SHAP: Shapley additive explanations
XGBoost: eXtreme Gradient Boosting

## Data Availability

According to Israel Ministry of Health regulations and Maccabi Healthcare Services policy, individual-level data cannot be shared openly (even if deidentified). Specific requests for remote access to deidentified community-level data will be reviewed by Kahn-Sagol-Maccabi, Maccabi Healthcare Services Research and Innovation Center.
